# Small-Angle X-Ray Scattering Reveals Compact Domain-Domain Interactions in the N-Terminal Region of Filamin C

**DOI:** 10.1371/journal.pone.0107457

**Published:** 2014-09-22

**Authors:** Ritika Sethi, Jari Ylänne

**Affiliations:** Department of Biological and Environmental Science and Nanoscience Center, University of Jyväskylä, Jyväskylä, Finland; University of Oulu, Finland

## Abstract

Filamins are multi-domain, actin cross-linking, and scaffolding proteins. In addition to the actin cross-linking function, filamins have a role in mechanosensor signaling. The mechanosensor function is mediated by domain-domain interaction in the C-terminal region of filamins. Recently, we have shown that there is a three-domain interaction module in the N-terminal region of filamins, where the neighboring domains stabilize the structure of the middle domain and thereby regulate its interaction with ligands. In this study, we have used small-angle X-ray scattering as a tool to screen for potential domain-domain interactions in the N-terminal region. We found evidence of four domain-domain interactions with varying flexibility. These results confirm our previous study showing that domains 3, 4, and 5 exist as a compact three domain module. In addition, we report interactions between domains 11–12 and 14–15, which are thus new candidate sites for mechanical regulation.

## Introduction

Filamins (FLNs) are a family of actin cross-linking proteins essential for the maintenance of cellular architecture and function [1]. They also act as scaffolds for many transmembrane, signaling, and cytoskeletal proteins [2]. There are three FLN genes in vertebrates. Filamin A (*FLNA*) and filamin B (*FLNB*) are expressed ubiquitously whereas filamin C (*FLNC*) is muscle specific [3]. FLNs are homodimers, and a single subunit of vertebrate FLN consists of an N-terminal actin-binding domain (ABD) followed by 24 immunoglobulin-like (Ig) domains. A typical FLN Ig domain consists of seven anti-parallel β -strands (A–G) that are arranged into two β sheets facing each other [2], [4]. Ig domains 1–15 are referred to as Rod 1 and 16–24 as Rod 2. A comparison of different FLN sequences shows a repeating pattern of two domains, suggesting that the protein has evolved via tandem duplications of two domains [5]. In line with this, a regular pattern of alternating calculated isoelectric points of neighboring domains can be observed in all three vertebrate FLN isoforms [6]. These features correlate with the existence of three closely interacting domain pairs in the Rod 2 region of FLNa: domains 16–17, 18–19 and 20–21 [7], [8]. In FLNa domains 18–19 and 20–21, the A strand of the even numbered domain is natively detached and forms an anti-parallel strand next to the strand C of the odd domain [7], [8]. These domain pairs function as mechanically regulated interaction sites for cell adhesion receptors and signaling adaptors, since the dissociation of the A strand of the odd domain from domains 19 and 21 can be regulated by low pico newton range mechanical forces [9]–[11]. This allows the interacting proteins to bind to the surface that is normally masked by the A strand [12], [13]. Thus, ligand binding in the Rod 2 region of FLNs is negatively regulated by inter-domain interactions. The existence of these regulated, compact domain pairs in the Rod 2 region of FLNa fits well with electron microscopy (EM) images showing that this fragment is globular. On the contrary, the Rod 1 region of FLNa is extended in EM images [14]. However, as protein interaction sites have also been observed in the Rod 1 region [15], it is possible that some of the interaction sites in this region are regulated by domain-domain interactions. Indeed, we have recently shown that in FLNa and FLNc, domains 3–5 form a compact structure where domain 5 stabilizes domain 4 and may in that way regulate the interactions of domain 4 [16].

In this study, we conduct a systematic small-angle X-ray scattering (SAXS) screen of all possible two-domain fragments of FLNc to search for domain-domain interactions in the Rod 1 region of FLNc. These results confirm the previously observed interactions between domains 3–4 and 4–5 and reveal two new interactions.

## Results

### Small-angle X-ray scattering analysis reveals four compact two-domain fragments

**Figure 1:**
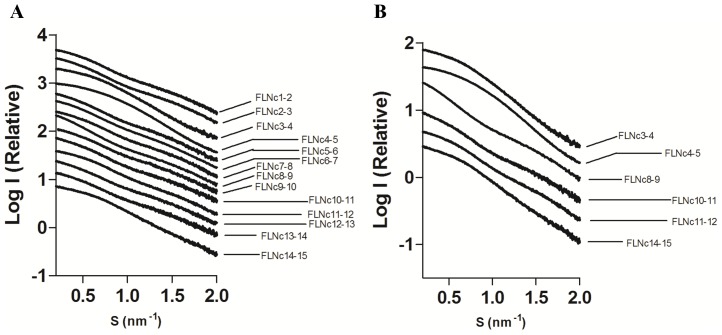
Logarithmic scattering curves as a function of momentum transfer (s). (**A**) Comparison of all the constructs studied. (**B**) Comparison of compact two-domain fragments with the average (FLNc10-11) and completely extended fragments (FLNc8-9). The curves are displaced on the Y-axis for clarity.

**Figure 2:**
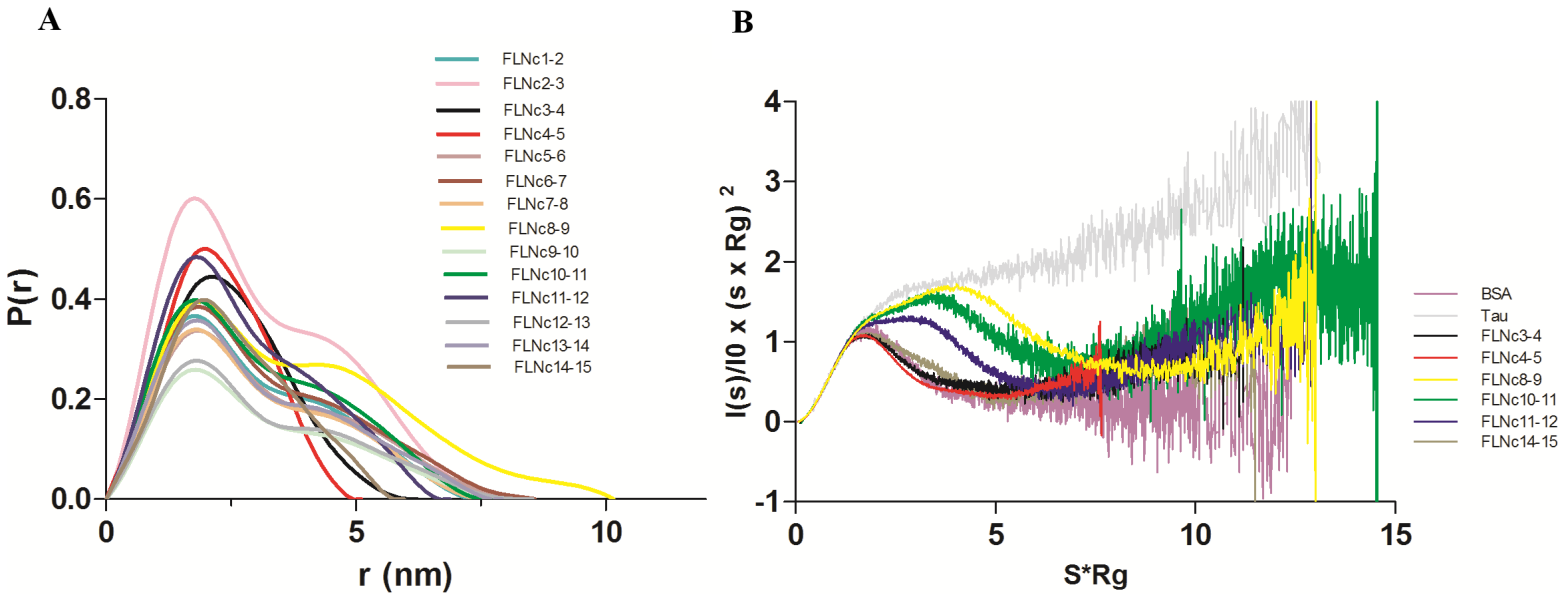
Distance distribution profile and dimensionless Kratky plots. (**A**) P(r) vs r plot of all the constructs. This is supported by Supporting Information S4 in File S1. (**B**) Dimensionless Kratky plots of the four compact, average extended and most extended fragments compared to BSA (folded protein) and Tau (unfolded protein).

SAXS is a low resolution structural technique that gives valuable information about the overall conformation and conformational changes of molecules in solution [17], [18]. It is a powerful tool for studying large multi-domain proteins, which may be difficult to crystallize because of their flexibility. To screen for compact tandem domain pairs in the Rod 1 region of FLNc, we cloned, expressed, and purified all 14 possible two-domain fragments from the Rod 1 of human FLNc. All the fragments could be purified to near homogeneity (at least 90–95%) and high molecular weight contaminants could be removed by the final gel filtration step of the purification procedure (Supporting Information S1 in File S1). The solution behavior of these constructs was analyzed using SAXS. The quality of scattering data was excellent throughout the protein concentration range used. There were no concentration-dependent changes in the low angle scattering or calculated radius of gyration (R_g_) (Supporting Information S2 in File S1), and the Guinier region analysis showed a linear fit, which was consistent with a lack of inter-particle interactions (Supporting Information S3 in File S1). Experimental scattering profiles of FLNc domains 3–4, 4–5 and 14–15 revealed features in the low angle range (Figure 1), suggesting that these constructs behave differently in solution from the rest of the two-domain fragments. SAXS-derived size-related parameters are summarized in Table 1. The average R_g_ (mean square distances from the center of mass weighted by electron densities) and D_max_ (maximum dimension of the particle) for the fragments was 2.3 and 7.7 nm respectively. Based on smaller than average values of D_max_ and R_g_, four fragments were compact, domains 3–4, 4–5, 11–12 and 14–15. Further, the shape of the pairwise distance distribution plot (P(r) vs r) for domains 4–5 showed a compact bell-shaped profile, suggesting this fragment was globular [19], [20] (Figure 2A, red curve). A similar but broader profile was visible in domains 3–4 (Figure 2A, black curve) and 14–15 (Figure 2A, brown curve). It is interesting to note that, although domains 11–12 had a smaller than average D_max_, the distance distribution plot showed a bimodal peak distribution, indicating some degree of flexibility between the domains (Figure 2A, blue curve). The rest of the two-domain fragments showed broad bimodal peak distributions and high D_max_ values (Figure 2A and Table 1). The fits of the P(r) function with the experimental data for all the fragments are shown in Supporting Information S4 in File S1. Taken together, these results imply that there are inter-domain interactions in the N-terminal Ig domains of FLN similar to the domain pairs found in the C terminus. They also imply that the fragment of FLNc domains 4 and 5 is the most compact.

**Table 1:** Parameters derived from SAXS measurements.

Protein	I(0) from Guinier	I(0) from P(r)	R_g_ (nm) from Guinier	R_g_ (nm) from P(r)	D_max_ (nm)$	Porod Volume (nm^3^)	Mw (kDa) estimation from sequence	Mw (kDa) estimation from I(0)	Mw (kDa) estimation from Porod Volume
FLNc1-2	16.34+−0.02	16.37	2.31+−0.02	2.33	7.6	25.26	21	18	15
FLNc2-3	27.65+−0.03	27.57	2.39+−0.01	2.40	8.0	25.32	21	30	15
FLNc3-4	16.13+−0.02	16.40	1.86+−0.01	1.92	6.2	27.51	20	17	16
FLNc4-5	16.00+−0.01	16.10	1.72+−0.01	1.73	5.1	27.72	20	15	16
FLNc5-6	16.02+−0.03	15.99	2.47+−0.02	2.51	8.5	26.75	21	17	16
FLNc6-7	18.01+−0.02	18.09	2.45+−0.03	2.52	8.6	28.74	21	17	17
FLNc7-8	15.04+−0.02	15.13	2.37+−0.03	2.35	7.8	25.24	21	16	15
FLNc8-9	23.28+−0.05	23.36	2.90+−0.11	3.00	10.1	28.41	19	22	17
FLNc9-10	11.84+−0.03	11.84	2.39+−0.23	2.42	8.2	24.25	19	13	14
FLNc10-11	19.05+−0.05	18.82	2.42+−0.05	2.39	7.8	24.09	21	20	14
FLNc11-12	19.98+−0.02	20.08	2.15+−0.01	2.17	6.9	25.05	20	21	15
FLNc12-13	12.72+−0.03	12.64	2.44+−0.02	2.45	8.5	26.33	20	14	15
FLNc13-14	16.23+−0.04	16.24	2.37+−0.03	2.40	7.9	25.53	20	17	15
FLNc14-15	14.42+−0.02	14.68	1.91+−0.04	1.97	6.0	31.23	21	15	18

$ The values written here are with an approximate error of 0.5 nm.

### Dimensionless Kratky and ensemble optimization method analysis reveals that FLNc domains 3–4, 4–5, and 14–15 are least flexible

To analyze the flexibility of the fragments, we represented the scattering data in a dimensionless Kratky plot [21] (Figure 2B), where we qualitatively compared the flexibility of the four compact fragments and the average extended and most extended fragments against well folded bovine serum albumin (BSA) [22] and fully unfolded Tau protein [23]. Once again, domains 3–4 (black), and 4–5 (red), and 14–15 (brown) showed sharp peaks similar to BSA (purple), suggesting that these fragments are globular. The fragment of domains 11–12 (blue), did not show a sharp peak, but was still comparatively less flexible than the average extended domains 10–11 (green) and fully extended domains 8–9 (yellow).

**Figure 3:**
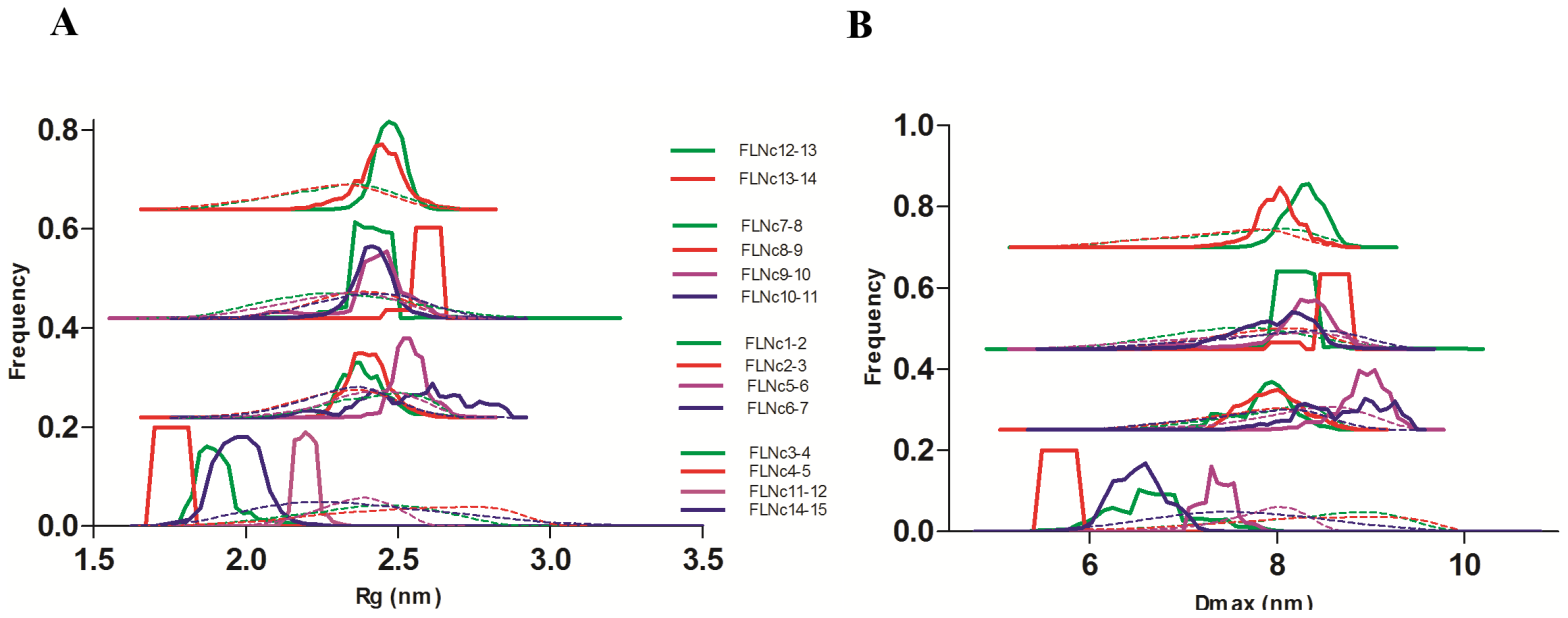
EOM analyses of all constructs. (**A**) R_g_ distribution profile. (**B**) D_max_ distribution profile. Dotted lines represent the distribution of the random pool, and solid lines show the distribution of the selected models. Top three panels are made by nudging the curves for clarity.

To gain further insight into the flexibility of all the two-domain fragments, we analyzed the data by the ensemble optimization method (EOM) [24] (Figure 3). The R_g_ and D_max_ distribution profiles of the selected ensembles (Figure 3, solid lines) of domains 3–4, 4–5, 11–12 and 14–15 showed a narrow range compared to the rest of the fragments. The average size-related parameters of the EOM-generated ensembles (Table 2) of these constructs were also similar to the parameters reported using the Guinier approach [25] (Table 1).

**Table 2:** Parameters derived from EOM analysis.

Protein	Average R_g_ (nm)	Average D_max_ (nm)
FLNc1-2	2.40	8.0
FLNc2-3	2.42	8.1
FLNc3-4	1.92	6.7
FLNc4-5	1.77	5.7
FLNc5-6	2.55	8.9
FLNc6-7	2.56	8.8
FLNc7-8	2.41	8.3
FLNc8-9	2.60	8.5
FLNc9-10	2.43	8.1
FLNc10-11	2.42	7.9
FLNc11-12	2.20	7.4
FLNc12-13	2.50	8.3
FLNc13-14	2.45	8.0
FLNc14-15	1.98	6.6

Hence, both dimensionless Kratky and EOM analyses suggested that the fragment of domains 4–5 was the least flexible, followed by domains 3–4 and 14–15. Domains 11–12 were at the borderline between partly and fully flexible two-domain fragments.

### SAXS-based modeling confirms the existence of compact regions

**Figure 4:**
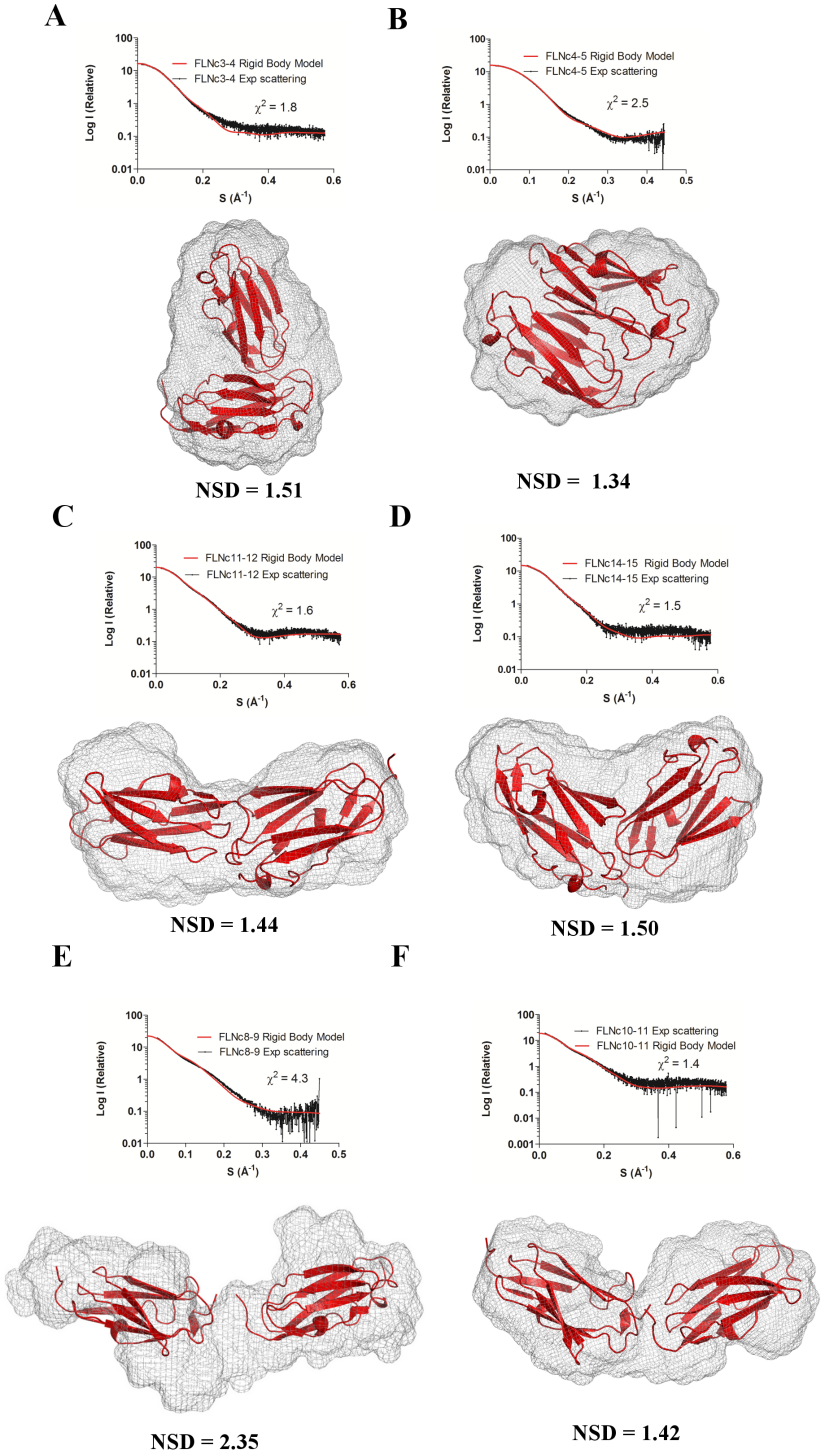
*Ab-initio* envelopes and rigid body models of selected two-domain fragments. The most compact two-domain fragments are domains 4–5 (**B**), 3–4 (**A**) and 14–15 (**D**); the fragment of domains 11–12 is intermediate (**C**). Domains 8–9 form the most extended two-domain fragment (**E**) and domains 10–11 is the average two-domain fragment (**F**). In the lower panels, the *ab-initio* and rigid body models are superimposed and their fits are expressed in terms of NSD. The fits of the rigid body models with the experimental scattering are given in the upper panels and the goodness of the fits is expressed in terms of X^2^. Please see Supporting Information S5 in File S1 for models of the rest of the fragments.

To visualize the shape of the two-domain fragments in solution, we derived three-dimensional models from the scattering data. *Ab-initio* envelopes (Figure 4 and Supporting Information S5 in File S1, grey mesh) that represent the overall shape of the molecule in solution, were generated using the GASBOR [26] and DAMAVER package [27] of ATSAS. They were then refined against the experimental scattering data using DAMMIN [28]. Once again, the shape of the envelope of domains 4–5 (Figure 4B) appeared to be most globular, followed by domains 14–15 (Figure 4D), and 3–4 (Figure 4A). The overall profile of the domains 11–12 (Figure 4C) envelope was similar to that of a partly extended two-domain fragment with maximum dimensions close to the average D_max_ (Figure 4F). The shape of the envelope of the most extended two-domain fragment (Figure 4E) was clearly more stretched.

We further generated rigid body models (Figure 4 and Supporting Information S5 in File S1, red cartoon) that best fit the experimental scattering (Figure 4 and Supporting Information S5 in File S1, upper panel) from each construct using SASREF [29]. The superposition of these models on the *ab-initio* envelopes (Figure 4 and Supporting Information S5 in File S1, grey mesh and red cartoon) suggested that domains 4 and 5 interacted with each other side-by-side and that one domain was tilted with respect to the other in domains 3–4 and 14–15. Domains 11–12 looked similar to the partly extended two-domain fragment of domains 10–11 with average dimensions. The sizes and shapes of all the other two-domain fragments fit to the elongated model where two domains interact only at their ends (Figure 4 E, F and Supporting Information S5 in File S1). These results indicated existence of inter-domain interactions in the N-terminal Ig domains of FLN similar to the domain pairs found in the C terminus.

## Discussion

Filamins are F-actin-binding proteins that also act as scaffolds for various signaling processes [2], [12]. Their multi-modular architecture, along with their association with the actin cytoskeleton, plasma membrane, and nuclear envelope [1], make them perfect candidates for mechanosensors [12]. For FLNs to convert mechanical cues to biochemical signals, they undergo conformational changes that regulate ligand binding by exposing or masking cryptic interaction sites [7]–[10]. Until recently, the Rod 2 region of FLNs has been identified as the major player of the mechanosensor function. On the contrary, the domains in Rod 1 are considered to have an extended domain arrangement [6], [14] and their function is not entirely clear. Studies have been conducted in the past in an attempt to understand the role of Rod 1 domains by comparing their sequences with the Rod 2 domains and then measuring their interactions. To this end, domains 4, 9, and 12 have been shown to interact with similar peptides as the Rod 2 domains [15]. To investigate the functions and regulation of the N-terminal Ig domains of FLNs, we probed consecutive domain-domain interactions in the Rod 1 region.

Based on previous results showing that the interactions were mediated by two-domain fragments in the Rod 2 region [7], [30], [31], we screened all possible two-domain fragments from the first 15 Ig-domains of FLNc. We found four compact pairs: domains 3–4, 4–5, 11–12 and 14–15 (Figure 1 and Table 1). The R_g_ and D_max_ values for three of these were similar to the previously characterized domain pairs at the Rod 2 region [7]. The finding that domains 3–4 and 4–5 were compact in this SAXS screening was in consensus with our recent crystal structures showing that domains 3, 4, and 5 formed a compact three-domain module (pdb codes 3V8O and 4M9P) [16]. We did not observe interactions between any other two-domain fragments in tandem; therefore, domains 11–12 and 14–15 are the new candidates whose potential for inter-domain interactions was brought forward in this study. Interestingly however, the scattering profiles of FLNc domains 3–4, 4–5 and 14–15 clearly looked distinguishable from the non-interacting two domain fragments, the scattering curve of domains 11–12, on the other hand, looked very similar to non-interacting fragments (Figure 1). The same held true for the dimensionless Kratky (Figure 2 B) and EOM analyses (Figure 3), where domains 11–12 seemed to be highly flexible. In addition, though the scattering-derived parameters, R_g_ and D_max_, showed it to be compact, the *ab-initio* and rigid body models showed that domains 11–12 were significantly extended. Therefore, while the fragment of domains 14–15 was clearly globular, the fragment of domains 11–12 was at the borderline between the compact and the fully extended two-domain fragments.

**Figure 5:**
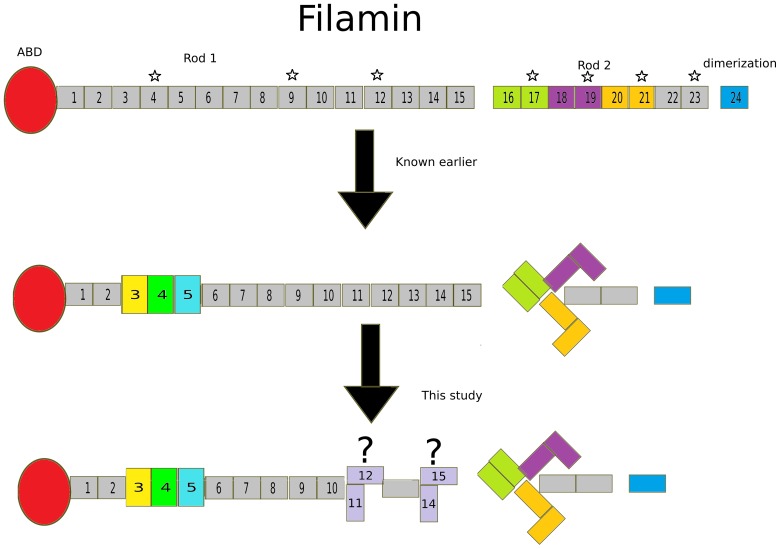
Summary of the results. **Each panel** shows a monomer of FLN with ABD in red, followed by 24 Ig repeats and dimerization domain in blue. **Upper panel** shows previously known domain pairs in the Rod 2 region, FLN 16–17, 18–19 and 20–21 in lemon, purple and mustard, respectively. The domains that share high sequence similarity with pair forming domain 21 are highlighted with a star. **Middle panel** shows the recently revealed three-domain module in the Rod 1 region (Domain 3 in yellow, 4 in green and 5 in cyan). In addition, the arrangement of the three-domain pairs of the Rod 2 region is depicted. **Lower panel** shows the new candidates for inter-domain interactions revealed in this study.

Based on similar interaction properties and the sequence similarity with the pair-forming domains in Rod 2 [5], [15], domains 4, 9, and 12 were the prime candidates where domain-domain interactions may be involved in the regulation of interactions (Figure 5, asterisks in the upper panel). We have recently reported that domain 4 is stabilized by interaction with the neighboring domains [16]. Here, we did not observe interactions with FLNc domain 9 with its neighbors. However, we obtained some evidence that domain 12 may interact with domain 11, but the fragment of domain 11–12 still seemed flexible. In addition, we found that domains 14 and 15 may interact with each other and may be a prime candidate for a new regulated interaction site. Apart from the general sequence similarity between domains 4 and 12 [15], we did not observe any specific sequence features to explain the new domain-domain interactions reported in this study (domains 11–12 and 14–15). These sites do not share the two proline residues in the intervening sequence that may help bend domains 4 and 5 close together (Data not show, PDB ID 3V8O and 4M9P). It is of interest that, while the compact domain-domain interactions in the Rod 2 region of FLN were observed in electron microscopic studies, those reported here for the Rod 1 region were not [14]. One explanation for this may be that while the pairs in Rod 2 are in tandem and form a compact 6-domain fragment [30], [31], the compact regions in Rod 1 may be interrupted by stretches of extended domain arrangements; thus, these compact regions could fall below the technical resolution limits of rotary shadowing EM. Our results are consistent with the EM studies [14], where no long-range domain-domain interactions were observed in Rod 1. On the other hand, in EM images, 8-domain fragments of FLNa1-8 and FLNa8-15 were of equal lengths [14]. Therefore, these results support our current finding that there are domain-domain interactions within the FLN 8-15 fragment in addition to the three domain module of domains 3-5 (within FLN 1-8). The interaction between FLN domains 14 and 15 is also of interest, because these domains are located immediately before the flexible hinge region, which is alternatively spliced and regulates the overall flexibility of the full FLN dimer [32].

It is interesting to note that there was some degree of flexibility in all the two-domain fragments, which was suggested by the normalized spatial discrepancy (NSD) values (Figure 4 and Supporting information S5 in File S1) and the broad curves (solid lines, Figure 3) observed in the EOM analysis. However, the average *ab-initio* envelopes represented even the most extreme ab initio models (Supporting information S6 in File S1) quite well. The relative stability of the non-interacting two-domain fragments may be attributed to the presence of short linkers between domains.

In conclusion, the inter-domain interactions in the Rod 2 region of FLNs have been shown to regulate ligand binding [8], [9]. We have recently shown that there are also domain-domain interactions in the Rod 1 region (i.e. the three-domain module of FLN 3–5), where the neighboring domains stabilize the structure of domain 4 and may thereby regulate ligand binding to the domain [16]. Here we show that in addition to this module, FLN domains 11–12 and 14–15 form interacting domain pairs that are candidate sites for regulated interactions.

## Experimental Procedures

### Protein Expression and Purification

For expression of recombinant protein in Escherichia coli BL21 Gold strain (Agilent Technologies), two-domain fragments of human FLNc cDNA (GenBank AJ012737) were cloned in the GST-fusion protein vector pGTVL1 (Structural Genomics Consortium, University of Oxford) [33]. The proteins were expressed at 37°C for 4 h in the presence of 0.4 mM Isopropyl β-D-1-thiogalactopyranoside (IPTG). The GST-fusion proteins were first purified using Glutathione Agarose 4B (Protino, Machery-Nagel) and the fusion partner was released with Tobacco Etch virus protease (Invitrogen, Life technologies). The final purification step was gel filtration with HiLoad 26/60 Superdex 75 column (GE Healthcare) in 100 mM NaCl, 1 mM dithiothreitol (DTT) and 20 mM Tris pH 7.5. The proteins were concentrated with Centriprep YM-10000 centrifugal concentration devices (Millipore).

### Small-Angle X-ray Scattering Measurements

The beamlines for SAXS data collection were BM29 at the European Synchrotron Research Facility (ESRF) Grenoble (Exposure time  = 2 seconds; temperature  = 20°C and X33 at European Molecular Biology Laboratory (EMBL) Hamburg (Exposure time  = 15 seconds; temperature  = 10°C). The details of the beamline setups and detectors are given in the publications [34], [35]. Ten frames were collected and then merged for each sample to check for radiation damage. Different protein dilutions in the range of 1–10 mg/ml were tested in the same purification buffer as above with 10 mM DTT added right before the data collection. Buffer subtractions were done with the PRIMUS program [36] of the ATSAS package. Noise due to the beam stop at extremely small angles was removed prior to scaling the scattering intensity (I) according to different solute concentrations. The forward scattering I(0) and the radius of gyration (R_g_) were calculated for each concentration with the program GUINIER [25], assuming that at very small angles (s× Rg <1.3), the scattering intensity is:



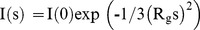
(1)


Based on the analysis of concentration-dependent changes and the Guinier region (Supporting Information S2 and S3 in File S1), the scattering data of the highest concentration was selected for each construct for further processing. The GNOM [37] program was used to calculate the distance distribution functions p(r) and the maximum particle dimensions D_max_ for all the fragments. The molecular mass of the constructs were evaluated by comparing the forward scattering from the fragments with that of reference solution of bovine serum albumin (BSA) with molecular mass, 66 kDa using the equation, Mw sample  = I(0) sample × Mw ref/I(0)ref. Porod's law was used to find out the excluded volume of the hydrated particle (*V*), assuming the samples were monodispersed:



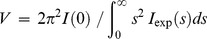
(2)


The same law was used to check the S^−4^ decay in scattering intensity at higher angles [38]. A dimensionless Kratky plot [21] (I(s)/I(0) × (s × Rg)^2^ versus s× Rg) was used to probe for the flexibility of the proteins compared to BSA [22] and Tau [23]. EOM was performed using the program EOM [24], wherein the sub- program RANCH generated a pool of 10,000 random models for each construct. The sub-program GAJOE then selected a set of representative models that best fit the experimental scattering. Ten rounds of GASBOR [26] were run to generate *ab-initio* envelopes using the scattering data. The chi values of each of the envelopes against the scattering data are tabulated in Supporting Information S7 in File S1. The envelopes were averaged with the program DAMAVER [27] to find the best envelope with common structural features. The averaged (NSD) of each model against all other models of the same construct is reported in a tabular form in Supporting Information S8 in File S1. DAMMIN [28] was used for the final model refinement against the scattering data. SASREF [29] was used to generate rigid body models using two chains of FLNc 23 (PDB ID: 2NQC) as the template with the distances of the N and C termini of the two domains defined. CRYSOL [39] was used to report the chi values of the fit of these models to the experimental data. The superposition of the averaged and refined *ab-initio* envelope with the respective rigid body model was done with SUPCOMB [40] and the figures were made using PyMOL (Schrödinger LLC, Portland, OR).

## Acknowledgments

We thank Ms. Arja Mansikkaviita for excellent technical assistance; Dr. Salla Ruskmao for help during data collection; and Jonne Seppälä and Dr. Ulla Pentikäinen for discussions. We are extremely thankful to Dr. Alexander V. Shkumatov for his valuable inputs and suggestions during the SAXS data analysis. We acknowledge European Molecular Biology Lab (EMBL) and European Synchrotron Radiation Facility (ESRF) for providing synchrotron access for the SAXS data collection. We express gratitude to Michal J. Gajda (EMBL Hamburg) and Matthew Bowler, Adam Round, and Petra Pernot (ESRF, Grenoble) for their assistance during the data collection at the beamlines.

## Supporting Information

File S1 **Supporting files. Supporting Information S1, Preparative size exclusion chromatography profiles of all the constructs studied.** (**A–N**): The y-axis is Absorbance at 280 nm wavelength and the x-axis is the elution volume in ml. In each purification, the main peak eluting between 150–200 ml was collected for further concentration and analysis. **Supporting Information S2, Check for concentration dependence of scattering.** (**A–N**): Logarithmic plot of scattering (I) versus momentum transfer (s) is shown for all concentrations of each construct. The low angle region is highlighted with a dotted box and the inset is shown on the upper right of each panel. R_g_ vs concentration plot for each construct is also shown on the bottom right of each panel. **Supporting Information S3, Guinier region representation of all the constructs.** (**A–N**): Experimental scattering in the low s range is shown with black dots. The fit is shown in red. The residuals points from the scattering curve that fall outside the Guinier fit are shown in green. **Supporting Information S4,** **Low angle fit of the reciprocal space scattering for the P(r) function analysis is shown (A–N). Supporting Information S5,** ***Ab-initio*** **envelopes and rigid body models of the rest of the two-domain fragments not shown in** **Figure 4:** ***Ab-initio*** **and rigid body models are superimposed and their fits are expressed in terms of NSD (A–H lower panel).** Fit of the rigid body model with the experimental data and the goodness of the fit (expressed in terms of X^2^) are given for each fragment (**A–H upper panel**). **Supporting Information S6, Superposition of the most typical (blue mesh) and the least typical (red mesh)** ***ab-initio*** **models with the averaged envelope (grey surface). Supporting Information S7, The chi values of each of the 10 GASBOR generated** ***ab-initio*** **models. Supporting Information S8, The average NSD of each of the 10 GASBOR generated** ***ab-initio*** **models.**
(DOCX)

## References

[1] Van Der Flier A, Sonnenberg A (2001) Structural and functional aspects of filamins. Biochim Biophys Acta 1538: 99–117. 11336782 10.1016/s0167-4889(01)00072-6

[2] Nakamura F, Stossel TP, Hartwig JH (2011) The filamins: organizers of cell structure and function. Cell Adh Migr 5: 160–169. 21169733 10.4161/cam.5.2.14401 PMC3084982

[3] Stossel TP, Condeelis J, Cooley L, Hartwig JH, Schleicher M, et al (2001) Filamins as integrators of cell mechanics and signalling. Nat Rev Mol Cell Biol 2: 138–145. 11252955 10.1038/35052082

[4] Pudas R, Kiema TR, Butler PJG, Stewart M, Ylänne J (2005) Structural basis for vertebrate filamin dimerization. Structure 13: 111–119. 15642266 10.1016/j.str.2004.10.014

[5] Light S, Sagit R, Ithychanda SS, Qin J, Elofsson A (2012) The evolution of filamin - a protein domain repeat perspective. J. Struct Biol 179: 289–298. 22414427 10.1016/j.jsb.2012.02.010 PMC3728663

[6] Djinovic-Carugo K, Carugo O (2010) Structural Portrait of Filamin Interaction Mechanisms. Curr Protein Pept Sci 11: 639–650. 20887254 10.2174/138920310794109111

[7] Heikkinen OK, Ruskamo S, Konarev PV, Svergun DI, Iivanainen T, et al (2009) Atomic structures of two novel immunoglobulin-like domain pairs in the actin cross-linking protein filamin. J Biol Chem 284: 25450–25458. 19622754 10.1074/jbc.M109.019661 PMC2757246

[8] Lad Y, Kiema T, Jiang P, Pentikäinen OT, Coles CH, et al (2007) Structure of three tandem filamin domains reveals auto-inhibition of ligand binding. EMBO J 26: 3993–4004. 17690686 10.1038/sj.emboj.7601827 PMC1948075

[9] Pentikäinen U, Ylänne J (2009) The regulation mechanism for the auto-inhibition of binding of human filamin A to integrin. J Mol Biol 393: 644–657. 19699211 10.1016/j.jmb.2009.08.035

[10] Ehrlicher AJ, Nakamura F, Hartwig JH, Weitz DA, Stossel TP (2011) Mechanical strain in actin networks regulates FilGAP and integrin binding to filamin A. Nature 478: 260–263. 21926999 10.1038/nature10430 PMC3204864

[11] Rognoni L, Stigler J, Pelz B, Ylanne J, Rief M (2012) Dynamic force sensing of filamin revealed in single-molecule experiments. Proc Natl Acad Sci 21: 1–6. 10.1073/pnas.1211274109 PMC3511698 23150587

[12] Razinia Z, Mäkelä T, Ylänne J, Calderwood DA (2012) Filamins in Mechanosensing and Signaling. Annu Rev Biophys 41: 227–246. 22404683 10.1146/annurev-biophys-050511-102252 PMC5508560

[13] Kiema T, Lad Y, Jiang P, Oxley CL, Baldassarre M, et al (2006) The Molecular Basis of Filamin Binding to Integrins and Competition with Talin. Mol Cell 21: 337–347. 16455489 10.1016/j.molcel.2006.01.011

[14] Nakamura F, Osborn TM, Hartemink CA, Hartwig JH, Stossel TP (2007) Structural basis of filamin A functions. J Cell Biol 179: 1011–1025. 18056414 10.1083/jcb.200707073 PMC2099194

[15] Ithychanda SS, Hsu D, Li H, Yan L, Liu DD, et al (2009) Identification and characterization of multiple similar ligand-binding repeats in filamin: implication on filamin-mediated receptor clustering and cross-talk. J Biol Chem 284: 35113–35121. 19828450 10.1074/jbc.M109.060954 PMC2787372

[16] Sethi R, Seppälä J, Tossavainen H, Ylilauri M, Ruskamo S, et al (2014) A Novel Structural Unit in the N-Terminal Region of Filamins. J Biol Chem 289: 8588–8598. 24469451 10.1074/jbc.M113.537456 PMC3961682

[17] Svergun DI, Koch MHJ (2002) Advances in structure analysis using small-angle scattering in solution. Curr Opin Struct Biol 12: 654–660. 12464319 10.1016/s0959-440x(02)00363-9

[18] Petoukhov MV, Svergun DI (2007) Analysis of X-ray and neutron scattering from biomacromolecular solutions. Curr Opin Struct Biol 17: 562–571. 17714935 10.1016/j.sbi.2007.06.009

[19] Putnam CD, Hammel M, Hura GL, Tainer JA (2007) X-ray solution scattering (SAXS) combined with crystallography and computation: defining accurate macromolecular structures, conformations and assemblies in solution. Q Rev Biophys 40: 191–285. 18078545 10.1017/S0033583507004635

[20] Jacques DA, Guss JM, Svergun DI, Trewhella J (2012) Publication guidelines for structural modelling of small-angle scattering data from biomolecules in solution. Acta Crystallogr D Biol Crystallogr 68: 620–626. 22683784 10.1107/S0907444912012073

[21] Durand D, Vivès C, Cannella D, Pérez J, Pebay-Peyroula E, et al (2010) NADPH oxidase activator p67(phox) behaves in solution as a multidomain protein with semi-flexible linkers. J Struct Biol 169: 45–53. 19723583 10.1016/j.jsb.2009.08.009

[22] Gazdag EM, Schöbel S, Shkumatov AV, Goody RS, Itzen A (2014) The structure of the N-terminal domain of the Legionella protein SidC. J Struct Biol (In Press). 10.1016/j.jsb.2014.02.003 24556577

[23] Shkumatov AV, Chinnathambi S, Mandelkow E, Svergun DI (2011) Structural memory of natively unfolded tau protein detected by small-angle X-ray scattering. Proteins 79: 2122–2131. 21560166 10.1002/prot.23033

[24] Bernadó P, Mylonas E, Petoukhov MV, Blackledge M, Svergun DI (2007) Structural Characterization of Flexible Proteins Using Small-Angle X-ray Scattering. J Am Chem Soc 129: 5656–5664. 17411046 10.1021/ja069124n

[25] Guinier A (1939) La diffraction des rayons X aux très petits angles: application à l'étude de phénomènes ultramicroscopiques. Ann Phys 12: 161–237.

[26] Svergun DI, Petoukhov MV, Koch MH (2001) Determination of domain structure of proteins from X-ray solution scattering. Biophys J 80: 2946–2953. 11371467 10.1016/S0006-3495(01)76260-1 PMC1301478

[27] Volkov VV, Svergun DI (2003) Uniqueness of ab initio shape determination in small-angle scattering. J Appl Cryst 36: 860–864. 10.1107/S0021889809000338 PMC5023043 27630371

[28] Svergun DI (1999) Restoring low resolution structure of biological macromolecules from solution scattering using simulated annealing. Biophys J 76: 2879–2886. 10354416 10.1016/S0006-3495(99)77443-6 PMC1300260

[29] Petoukhov MV, Svergun DI (2005) Global rigid body modeling of macromolecular complexes against small-angle scattering data. Biophys J 89: 1237–1250. 15923225 10.1529/biophysj.105.064154 PMC1366608

[30] Ruskamo S, Gilbert R, Hofmann G, Jiang P, Campbell ID, et al (2012) The C-terminal rod 2 fragment of filamin A forms a compact structure that can be extended. Biochem J 446: 261–269. 22676060 10.1042/BJ20120361

[31] Tossavainen H, Koskela O, Jiang P, Ylänne J, Campbell ID, et al (2012) Model of a six immunoglobulin-like domain fragment of filamin A (16–21) built using residual dipolar couplings. J Am Chem Soc 134: 6660–6672. 22452512 10.1021/ja2114882

[32] Gardel ML, Nakamura F, Hartwig JH, Crocker JC, Stossel TP, et al (2006) Prestressed F-actin networks cross-linked by hinged filamins replicate mechanical properties of cells. Proc Natl Acad Sci U S A 103: 1762–1767. 16446458 10.1073/pnas.0504777103 PMC1413620

[33] Gileadi O, Burgess-brown NA, Colebrook SM, Berridge G, Savitsky P, et al (2007) High Throughput Production of Recombinant Human Proteins for Crystallography. Methods Mol Biol 426: 221–246. 10.1007/978-1-60327-058-8_14 18542867

[34] Pernot P, Round A, Barrett R, De Maria Antolinos A, Gobbo A, et al (2013) Upgraded ESRF BM29 beamline for SAXS on macromolecules in solution. J Synchrotron Radiat 20: 660–664. 23765312 10.1107/S0909049513010431 PMC3943554

[35] Roessle MW, Klaering R, Ristau U, Robrahn B, Jahn D, et al (2007) Upgrade of the small-angle X-ray scattering beamline X33 at the European Molecular Biology Laboratory, Hamburg. J Appl Crystallogr 40: s190–s194.

[36] Konarev PV, Volkov VV, Sokolova AV, Koch MHJ, Svergun DI (2003) PRIMUS: a Windows PC-based system for small-angle scattering data analysis. J Appl Crystallogr 36: 1277–1282.

[37] Svergun DI (1992) Determination of the regularization parameter in indirect-transform. J Appl Cryst 25: 495–503.

[38] Rambo RP, Tainer JA (2011) Characterizing flexible and intrinsically unstructured biological macromolecules by SAS using the Porod-Debye law. Biopolymers 95: 559–571. 21509745 10.1002/bip.21638 PMC3103662

[39] Svergun D, Barberato C, Koch MHJ (1995) CRYSOL – a Program to Evaluate X-ray Solution Scattering of Biological Macromolecules from Atomic Coordinates. J Appl Cryst 28: 768–773.

[40] Kozin MB, Svergun DI (2001) Automated matching of high- and low-resolution structural models. J Appl Crystallogr 34: 33–41.

