# Supplementary material for: Small-Angle X-Ray Scattering Reveals Compact Domain-Domain Interactions in the N-Terminal Region of Filamin C
Source: PLoS One. 2014 Sep 22;9(9):e107457. doi: 10.1371/journal.pone.0107457 (PMC4170960; doi:10.1371/journal.pone.0107457)
Supplement: File S1 — Supporting files. Supporting Information S1, Preparative size exclusion chromatography profiles of all the constructs studied. (A–N): The y-axis is Absorbance at 280 nm wavelength and the x-axis is the elution volume in ml. In each purification, the main peak eluting between 150–200 ml was collected for further concentration and analysis. Supporting Information S2, Check for concentration dependence of scattering. (A–N): Logarithmic plot of scattering (I) versus momentum transfer (s) is shown for all concentrations of each construct. The low angle region is highlighted with a dotted box and the inset is shown on the upper right of each panel. Rg vs concentration plot for each construct is also shown on the bottom right of each panel. Supporting Information S3, Guinier region representation of all the constructs. (A–N): Experimental scattering in the low s range is shown with black dots. The fit is shown in red. The residuals points from the scattering curve that fall outside the Guinier fit are shown in green. Supporting Information S4, Low angle fit of the reciprocal space scattering for the P(r) function analysis is shown (A–N). Supporting Information S5, Ab-initio envelopes and rigid body models of the rest of the two-domain fragments not shown in Figure 4: Ab-initio and rigid body models are superimposed and their fits are expressed in terms of NSD (A–H lower panel). Fit of the rigid body model with the experimental data and the goodness of the fit (expressed in terms of X2) are given for each fragment (A–H upper panel). Supporting Information S6, Superposition of the most typical (blue mesh) and the least typical (red mesh) ab-initio models with the averaged envelope (grey surface). Supporting Information S7, The chi values of each of the 10 GASBOR generated ab-initio models. Supporting Information S8, The average NSD of each of the 10 GASBOR generated ab-initio models. (DOCX) [file pone.0107457.s001.docx]

**SUPPORTING INFORMATION FILE S1**

**
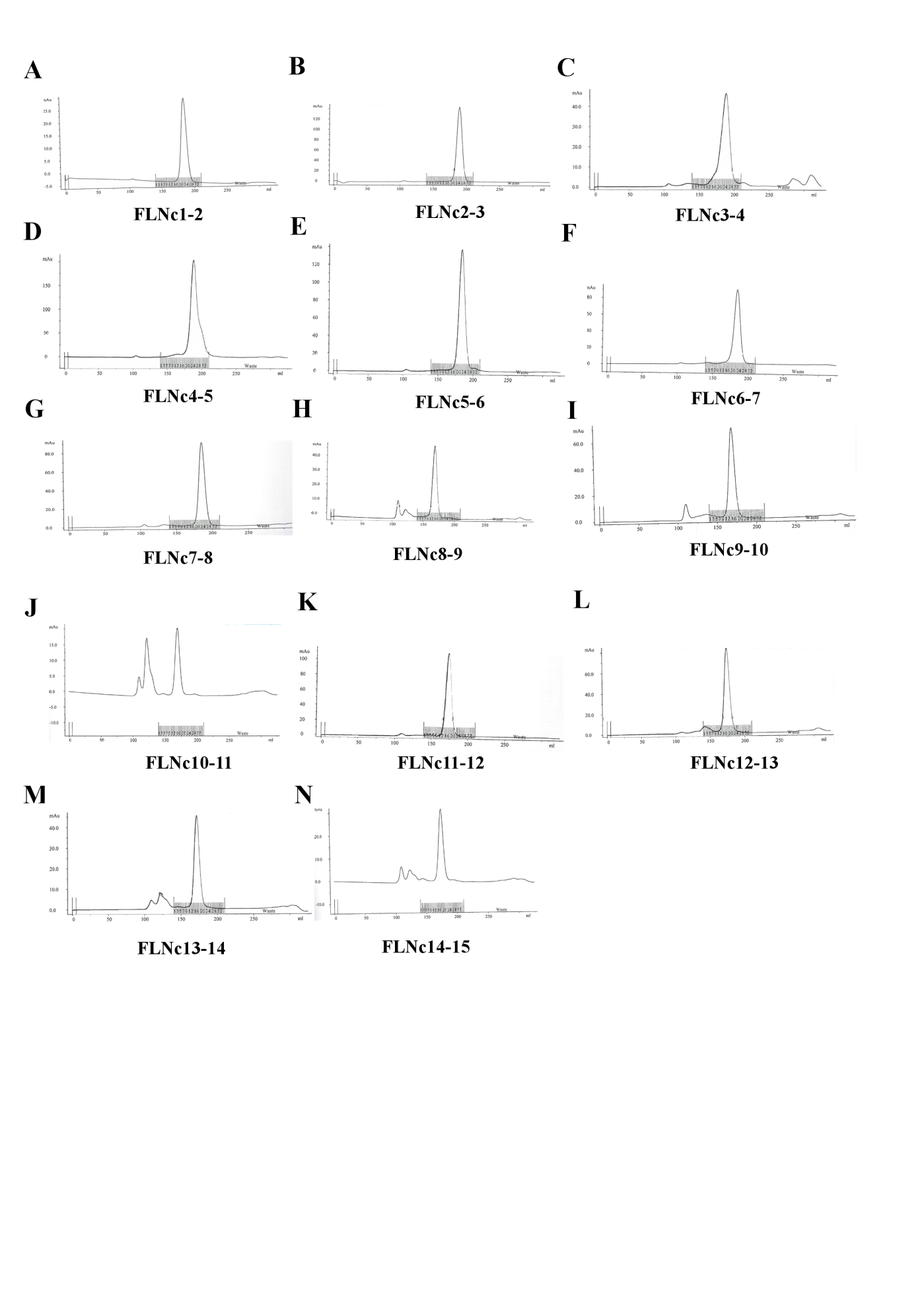
**

**Supporting Information S1** Preparative size exclusion chromatography profiles of all the constructs studied **(A-N)**: The y-axis is Absorbance at 280 nm wavelength and the x-axis is the elution volume in ml. In each purification, the main peak eluting between 150-200 ml was collected for further concentration and analysis.


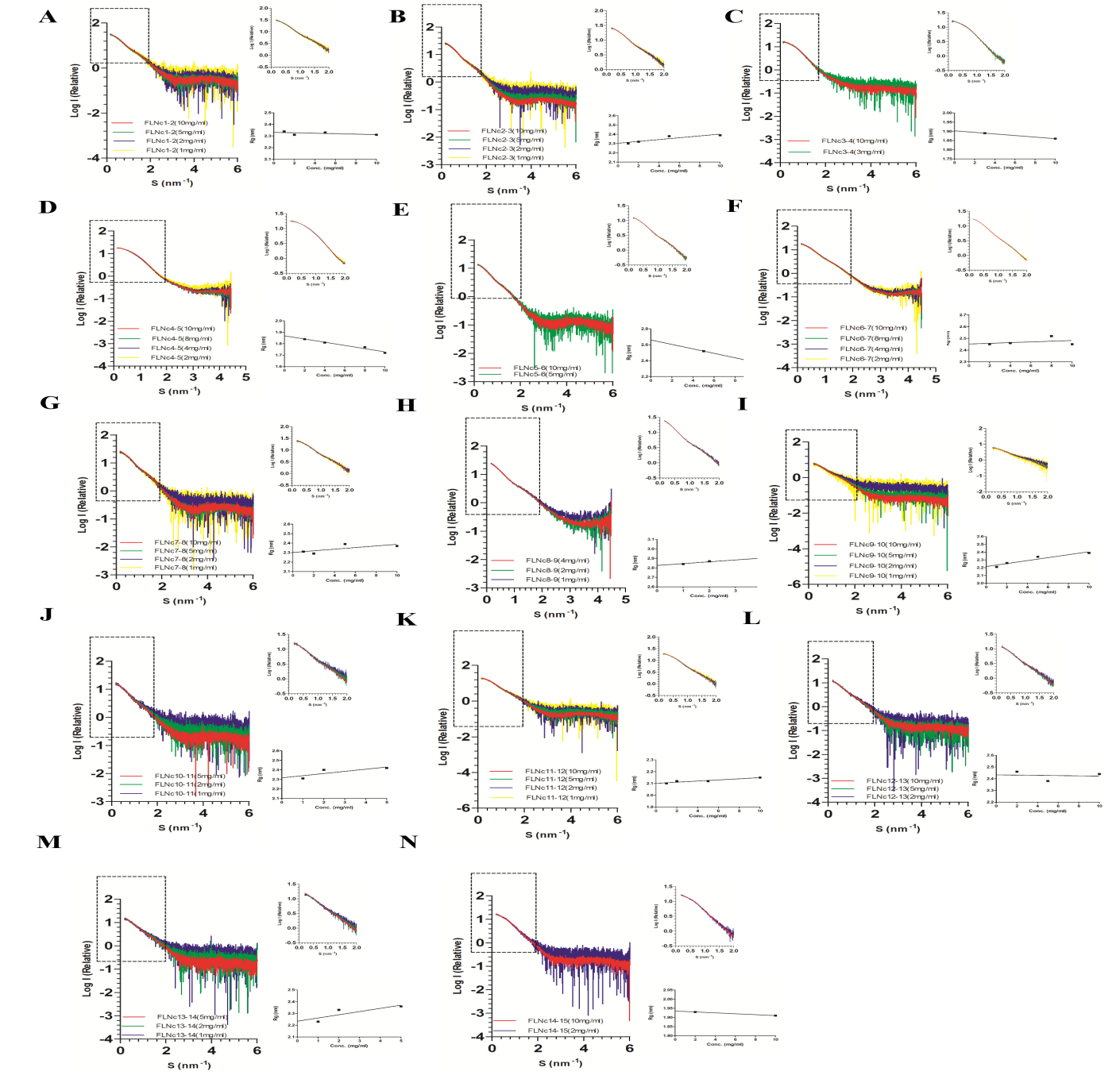


**Supporting Information S2** Check for concentration dependence of scattering **(A-N)**: Logarithmic plot of scattering (I) versus momentum transfer (s) is shown for all concentrations of each construct. The low angle region is highlighted with a dotted box and the inset is shown on the upper right of each panel. R_g_ vs concentration plot for each construct is also shown on the bottom right of each panel.


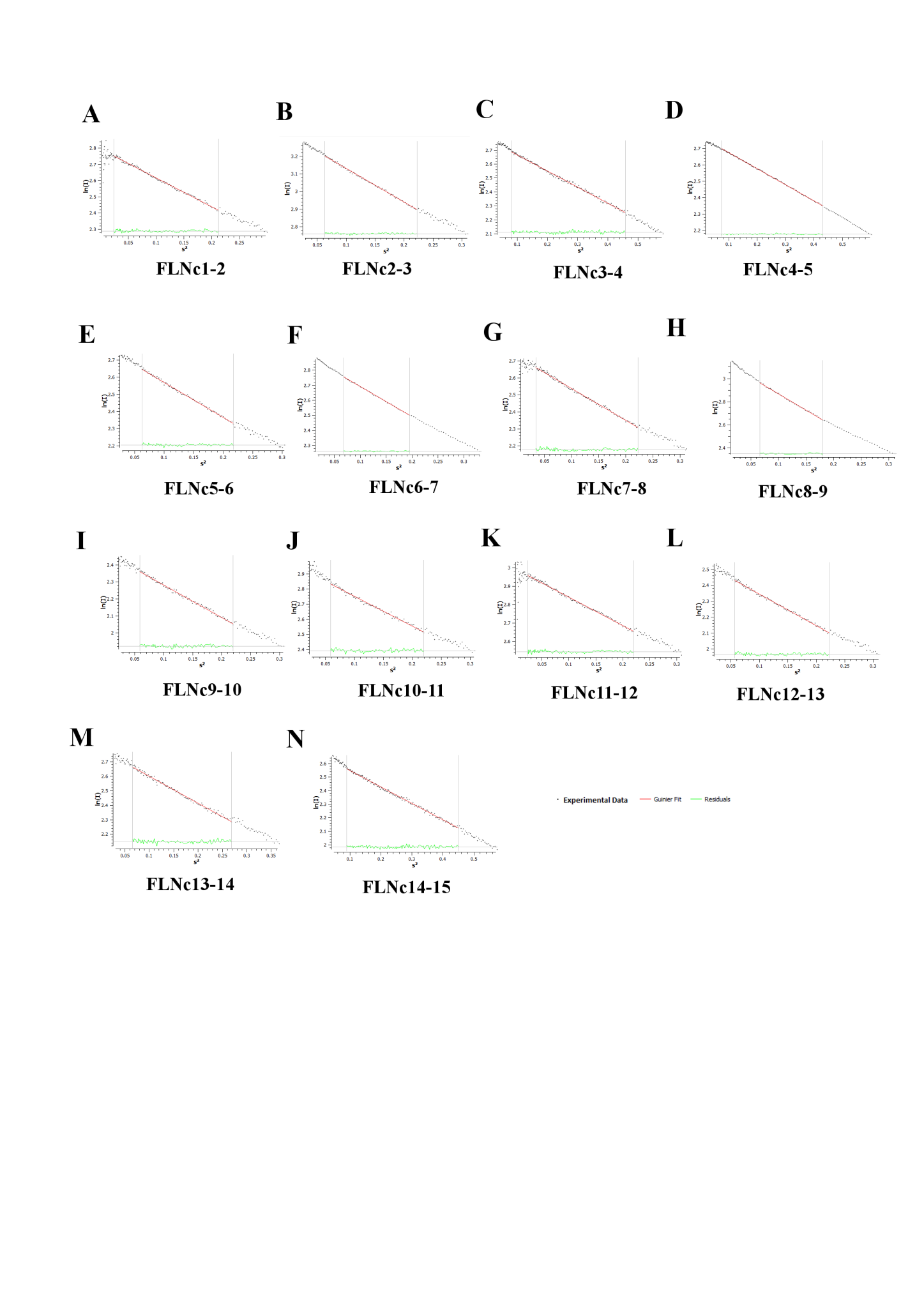


**Supporting Information S3** Guinier region representation of all the constructs **(A-N)**: Experimental scattering in the low s range is shown with black dots. The fit is shown in red. The residuals points from the scattering curve that fall outside the Guinier fit are shown in green.


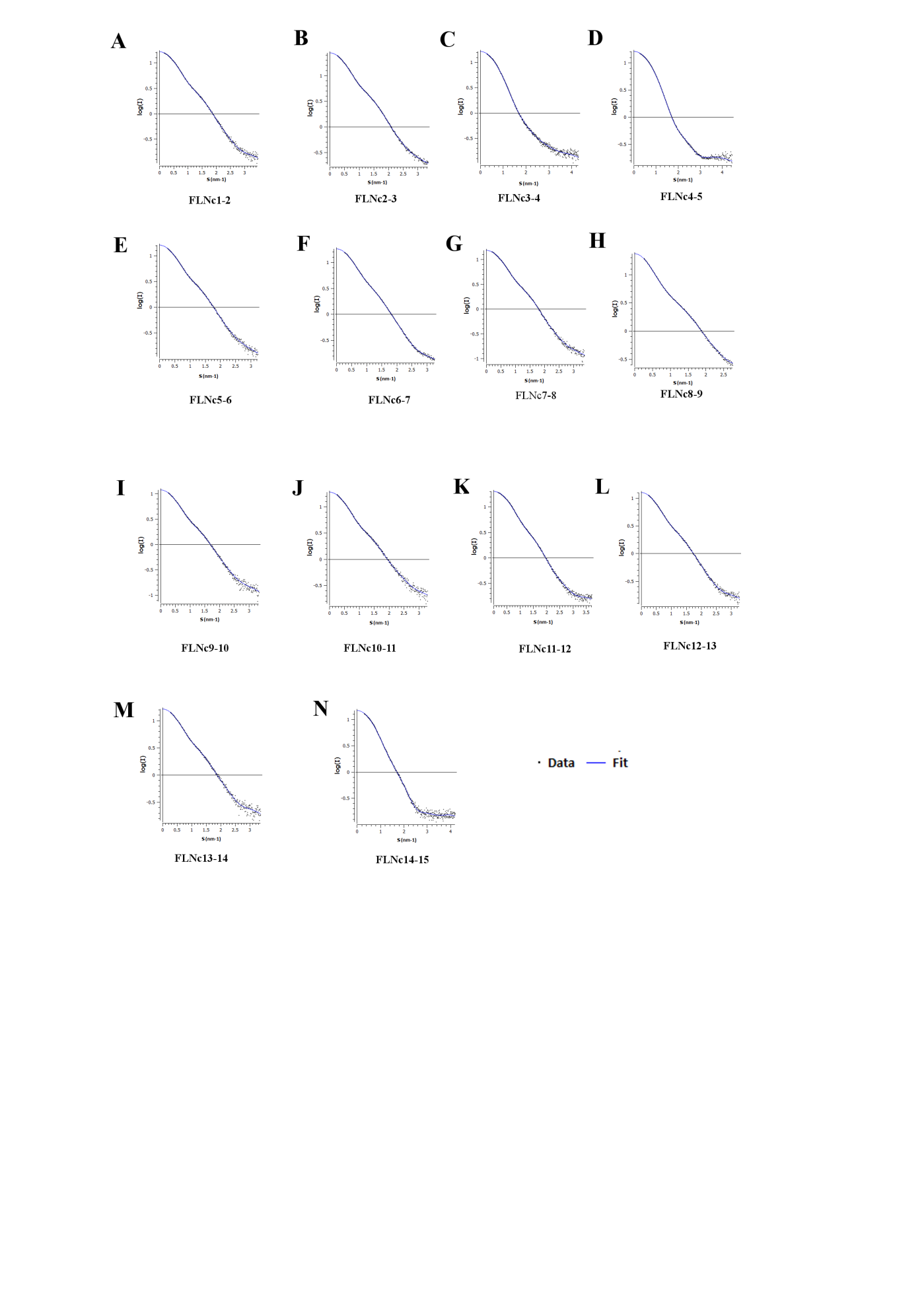


**Supporting Information S4** Low angle fit of the reciprocal space scattering for the P(r) function analysis is shown **(A-N)**.


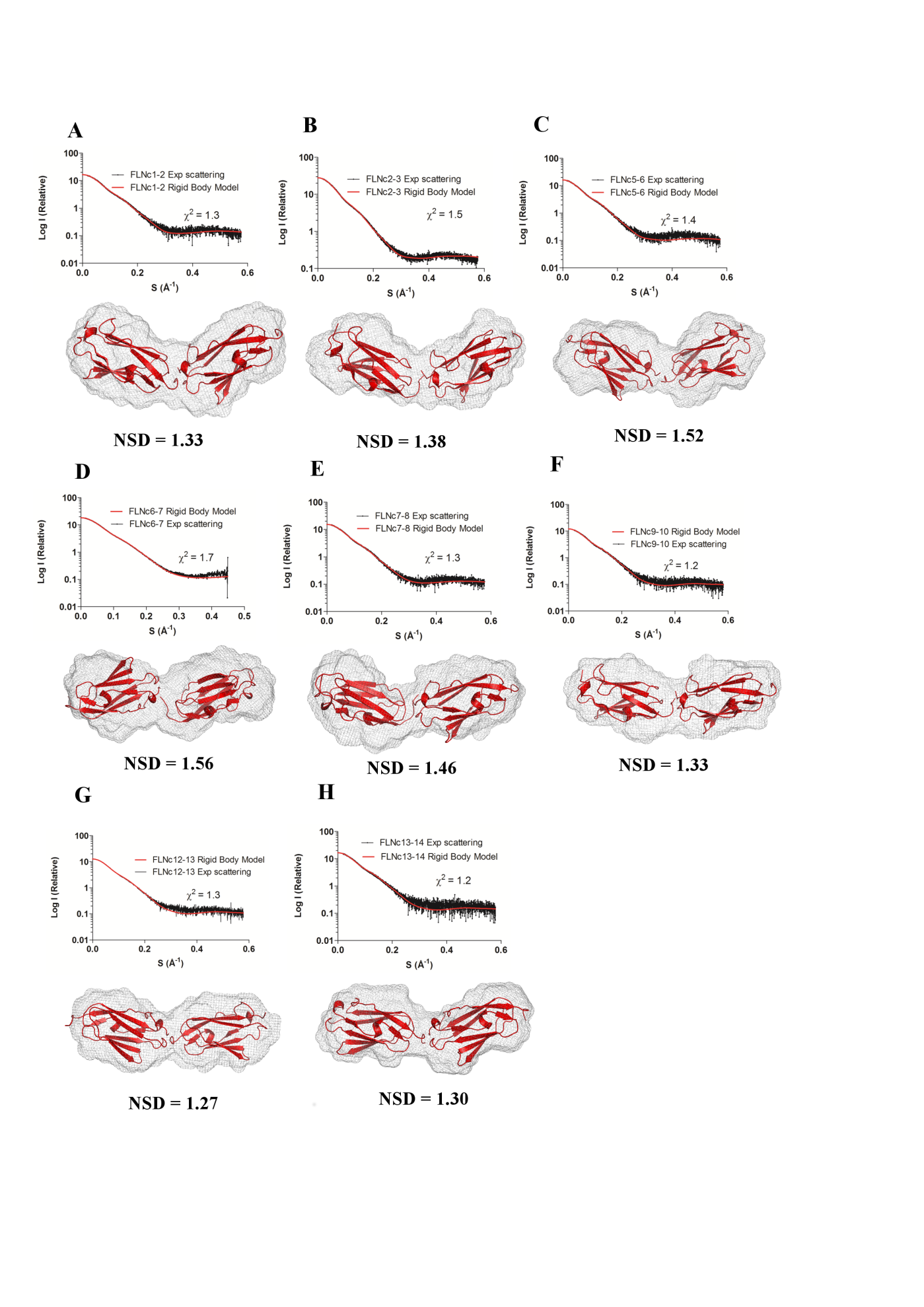


**Supporting Information S5** *Ab-initio* envelopes and rigid body models of the rest of the two-domain fragments not shown in Figure 4: *Ab-initio* and rigid body models are superimposed and their fits are expressed in terms of NSD **(A- H lower panel)**. Fit of the rigid body model with the experimental data and the goodness of the fit (expressed in terms of Χ^2^) are given for each fragment **(A-H upper panel)**.


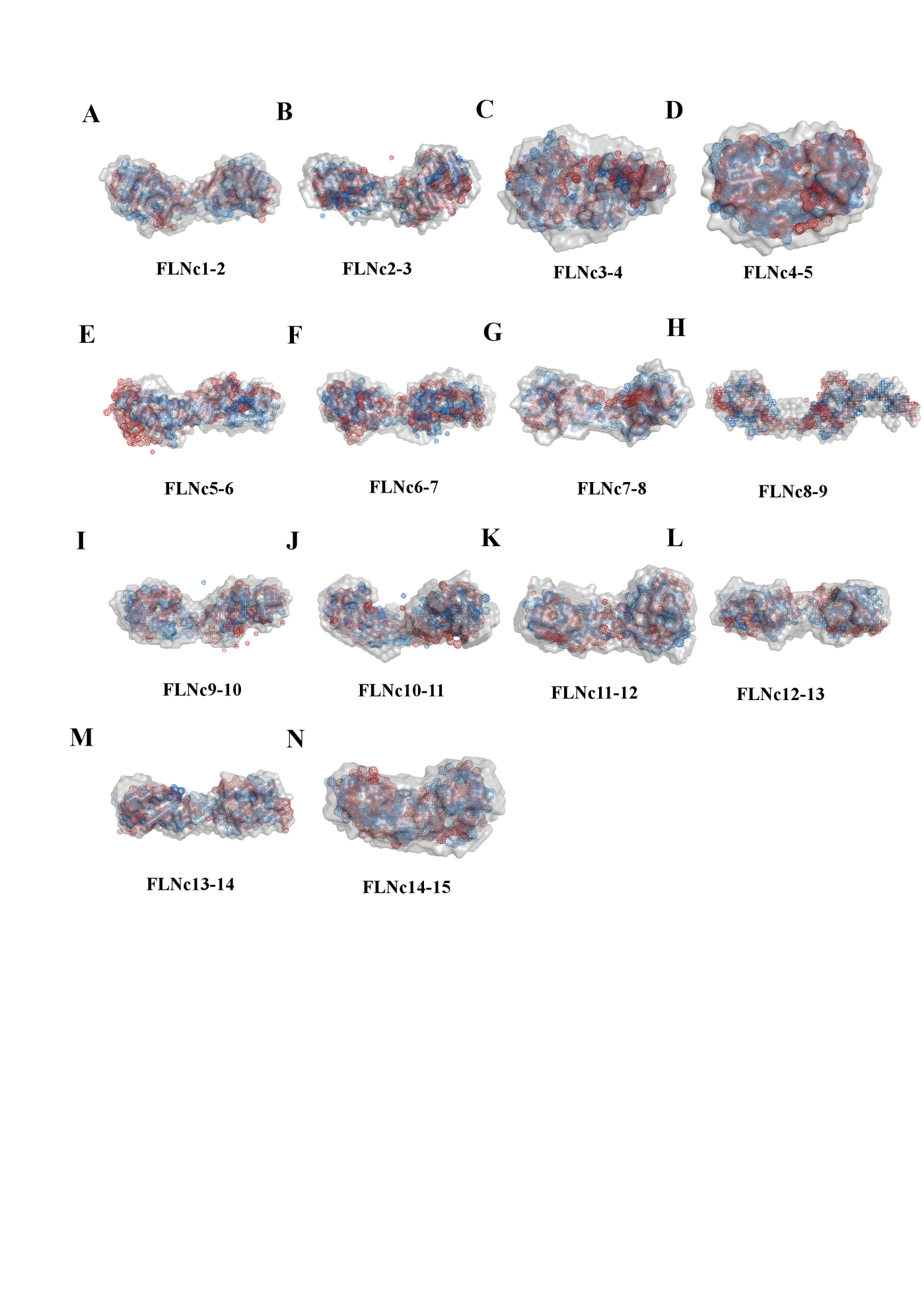


**Supporting Information S6** Superposition of the most typical (blue mesh) and the least typical (red mesh) *ab-initio* models with the averaged envelope (grey surface).

**Supporting Information S7:** The chi values of each of the 10 GASBOR generated *ab-initio* models.

| **FLNc1-2** | **FLNc2-3** | **FLNc3-4** | **FLNc4-5** | **FLNc5-6** | **FLNc6-7** | **FLNc7-8** |
| --- | --- | --- | --- | --- | --- | --- |
| 2.05 | 2.47 | 1.78 | 5.12 | 1.77 | 3.08 | 2.61 |
| 2.26 | 2.50 | 1.70 | 4.59 | 1.61 | 3.08 | 1.90 |
| 2.43 | 1.85 | 1.62 | 4.70 | 1.54 | 2.62 | 2.17 |
| 2.07 | 2.00 | 1.88 | 6.21 | 1.47 | 1.91 | 2.10 |
| 1.82 | 1.81 | 1.69 | 5.22 | 1.58 | 2.18 | 1.91 |
| 2.20 | 2.25 | 1.93 | 5.36 | 1.53 | 1.61 | 1.59 |
| 2.27 | 2.21 | 1.68 | 3.86 | 1.38 | 2.26 | 2.40 |
| 2.27 | 2.43 | 1.70 | 3.07 | 1.56 | 4.53 | 1.90 |
| 2.08 | 1.70 | 1.82 | 4.29 | 1.79 | 3.57 | 1.98 |
| 2.01 | 1.60 | 1.63 | 5.29 | 1.81 | 3.40 | 2.64 |

| **FLNc8-9** | **FLNc9-10** | **FLNc10-11** | **FLNc11-12** | **FLNc12-13** | **FLNc13-14** | **FLNc14-15** |
| --- | --- | --- | --- | --- | --- | --- |
| 1.46 | 1.81 | 1.64 | 2.12 | 1.36 | 1.33 | 1.88 |
| 1.67 | 1.19 | 1.48 | 2.12 | 1.41 | 1.11 | 1.87 |
| 1.70 | 2.14 | 1.44 | 2.92 | 1.61 | 1.19 | 1.80 |
| 1.20 | 1.79 | 1.50 | 2.14 | 1.40 | 1.65 | 1.63 |
| 1.88 | 1.73 | 1.44 | 2.62 | 1.62 | 1.22 | 1.85 |
| 1.37 | 1.58 | 1.20 | 2.49 | 1.60 | 1.43 | 1.83 |
| 2.00 | 1.37 | 1.46 | 2.42 | 1.45 | 1.19 | 1.69 |
| 1.60 | 1.69 | 1.05 | 2.65 | 1.86 | 1.03 | 1.75 |
| 1.71 | 1.75 | 1.41 | 2.78 | 1.28 | 1.67 | 1.62 |
| 1.40 | 1.42 | 1.33 | 2.99 | 1.30 | 1.45 | 1.95 |

**Supporting Information S8:** The average NSD of each of the 10 GASBOR generated *ab-initio* models.

| **FLNc1-2** | **FLNc2-3** | **FLNc3-4** | **FLNc4-5** | **FLNc5-6** | **FLNc6-7** | **FLNc7-8** |
| --- | --- | --- | --- | --- | --- | --- |
| 0.773 | 0.812 | 0.850 | 0.743 | 0.817 | 0.806 | 0.821 |
| 0.776 | 0.812 | 0.856 | 0.748 | 0.825 | 0.809 | 0.821 |
| 0.785 | 0.814 | 0.857 | 0.749 | 0.834 | 0.822 | 0.821 |
| 0.790 | 0.815 | 0.860 | 0.749 | 0.839 | 0.822 | 0.824 |
| 0.797 | 0.824 | 0.872 | 0.751 | 0.841 | 0.838 | 0.830 |
| 0.804 | 0.831 | 0.877 | 0.755 | 0.843 | 0.841 | 0.836 |
| 0.805 | 0.833 | 0.881 | 0.756 | 0.844 | 0.845 | 0.838 |
| 0.806 | 0.838 | 0.902 | 0.757 | 0.847 | 0.850 | 0.843 |
| 0.815 | 0.838 | 0.912 | 0.759 | 0.849 | 0.852 | 0.844 |
| 0.817 | 0.838 | 0.914 | 0.768 | 0.884 | 0.858 | 0.869 |

| **FLNc8-9** | **FLNc9-10** | **FLNc10-11** | **FLNc11-12** | **FLNc12-13** | **FLNc13-14** | **FLNc14-15** |
| --- | --- | --- | --- | --- | --- | --- |
| 1.013 | 0.823 | 0.794 | 0.780 | 0.782 | 0.776 | 0.797 |
| 1.028 | 0.828 | 0.799 | 0.785 | 0.785 | 0.786 | 0.798 |
| 1.029 | 0.833 | 0.801 | 0.792 | 0.789 | 0.788 | 0.801 |
| 1.040 | 0.836 | 0.804 | 0.804 | 0.791 | 0.798 | 0.803 |
| 1.043 | 0.843 | 0.819 | 0.805 | 0.793 | 0.803 | 0.804 |
| 1.091 | 0.846 | 0.828 | 0.807 | 0.795 | 0.806 | 0.805 |
| 1.100 | 0.848 | 0.829 | 0.819 | 0.797 | 0.808 | 0.806 |
| 1.115 | 0.855 | 0.829 | 0.830 | 0.797 | 0.811 | 0.812 |
| 1.130 | 0.856 | 0.834 | 0.857 | 0.799 | 0.811 | 0.818 |
| 1.164 | 0.869 | 0.850 | 0.857 | 0.816 | 0.824 | 0.850 |
